# Early gastric cancer metastasizing to the rectum, possibly via a hematogenous route: a case report and review of literature

**DOI:** 10.1186/s40792-016-0180-3

**Published:** 2016-06-07

**Authors:** Norio Uemura, Junji Kurashige, Keisuke Kosumi, Masaaki Iwatsuki, Kohei Yamashita, Shiro Iwagami, Yoshifumi Baba, Yasuo Sakamoto, Yuji Miyamoto, Naoya Yoshida, Yumi Honda, Yoshiki Mikami, Hideo Baba

**Affiliations:** Department of Gastroenterological Surgery, Graduate School of Medical Science, Kumamoto University, 1-1-1 Honjo, Chuo-ku, Kumamoto, 860-8556 Japan; Department of Diagnostic Pathology, Kumamoto University Hospital, Kumamoto, Japan

**Keywords:** Gastric cancer, Rectal recurrence, Hematogenous or lymphatic spread

## Abstract

**Background:**

The most common pattern of recurrence of gastric cancer (GC) is peritoneal dissemination. However, rectal metastasis via hematogenous or lymphatic spread is exceedingly rare. We present a case of a 65-year-old man with an intramucosal GC who developed a rectal recurrence, possibly via a hematogenous route.

**Case presentation:**

A 65-year-old man underwent curative endoscopic submucosal dissections for the intramucosal GCs at the anterior wall of the fornix twice. The third GC at the similar location was treated by radical laparoscopic proximal gastrectomy; microscopic examination revealed well-differentiated tubular adenocarcinoma confined to the lamina propria mucosae (T1aN0M0, stage IA). Follow-up colonoscopy revealed a 30-mm submucosal mass in the rectal wall 2 years later, and a metastasis of gastric origin was suspected histopathologically. After a staging laparoscopy confirmed the absence of findings suggestive of serosal involvement or peritoneal dissemination, including negative peritoneal washing cytology, a laparoscopic low anterior resection with lymph node dissection was performed. Microscopically, the tumor was found to mainly be located in the submucosal layer and showed features of moderately differentiated tubular adenocarcinoma. The serosal surface was free of disseminated tumor. Lymph node metastases were identified. Immunohistochemically, there were foci of carcinoma cells that were positive for cytokeratin 20; however, they were negative for cytokeratin 7. Negative staining for caudal-type homeobox 2, a transcription factor indicating goblet cell differentiation, combined with absence of intramucosal carcinoma in the rectal mucosa, suggested a diagnosis of metastatic adenocarcinoma of gastric origin. The absence of evidence of peritoneal dissemination suggested hematogenous or lymphatic spread.

**Conclusion:**

Although rectal metastasis from GC, particularly when attributable to hematologic or lymphatic metastasis, is very rare, metastatic gastric adenocarcinoma should be considered as a differential diagnosis for patients who present with a rectal tumor and a past history of GC, even if it is an early GC.

## Background

Gastric cancer (GC) is the fourth most commonly diagnosed cancer and the second most common cause of cancer mortality worldwide [1]. Despite improvements in diagnosis and treatment, the prognosis of patients with recurrent GC remains poor [2, 3], the recurrence rate after curative surgery for GC reportedly ranging from 20 to 50 % [4–8].

Based on the findings of pertinent studies, the recurrence patterns after curative surgery for GC have been classified as follows: (1) locoregional recurrence, (2) peritoneal recurrence, and (3) distant (including hematogenous) metastasis [3, 6, 9–11]. Of these, metastasis of GC to the colorectal region by hematogenous or lymphatic spread is very rare. There are a few English language reports of such cases [12–16], all of which had advanced-stage disease. We report a case of a metachronous rectal metastasis caused by hematologic or lymphatic spread 2 years after curative resection of a recurrent intramucosal GC.

## Case presentation

**Fig. 1:**
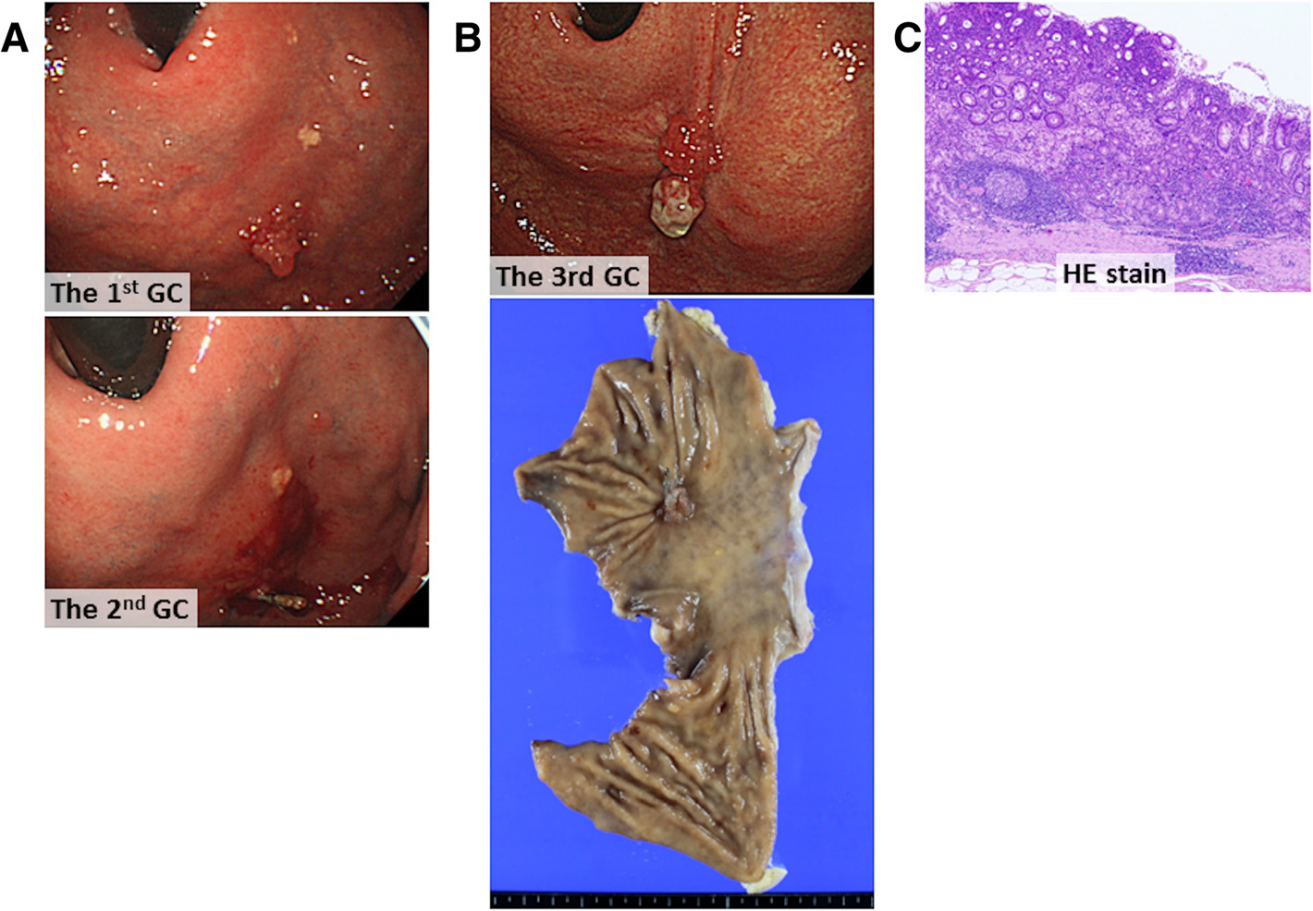
Findings of resected gastric cancers. **a** Gastroscopic images showing the first (*upper*) and second (*lower*) proximal gastric cancers. **b** Gastroscopic image of the third proximal gastric cancer (*upper*) and the macroscopic appearance of the resected proximal gastric specimen (*lower*). **c** Photomicrograph of a section from the resected gastric cancer showing well-differentiated tubular adenocarcinoma (hematoxylin and eosin stain, ×40)

A 60-year-old man underwent endoscopic submucosal dissection (ESD) for early GC at the anterior wall of the fornix (Fig. 1a, upper). Microscopic examination of the removed tissue established a diagnosis of well-differentiated tubular adenocarcinoma of intestinal type (Lauren classification) [17], which was confined to the lamina propria mucosae (pT1a, the American Joint Committee on Cancer classification seventh edition). No ulcer scar or lymphovascular invasion was identified, and the surgical margins were free of tumor.

Thirteen months later, he underwent the second ESD for the second early GC at the similar location (Fig. 1a, lower), which was confirmed to be an intramucosal well-differentiated tubular adenocarcinoma; the resection margin was negative for tumor. Again, no ulcer scar or lymphovascular invasion was identified. A perforation that occurred during ESD was closed immediately by clipping and was treated conservatively.

Three years after the second curative ESD, he underwent laparoscopic proximal gastrectomy with lymph node dissection for the third GC at the similar location (Fig. 1b, upper and lower). The adjacent non-neoplastic mucosa showed atrophic changes, and microscopic examination of the specimen revealed well-differentiated tubular adenocarcinoma, which was still confined to the lamina propria (Fig. 1c). The morphologic features of this tumor differed from those of the first and second tumors; this one was diagnosed as being of gastric type. Neither lymphatic nor venous invasion was identified, and the surgical margins were free of tumor.

**Fig. 2:**
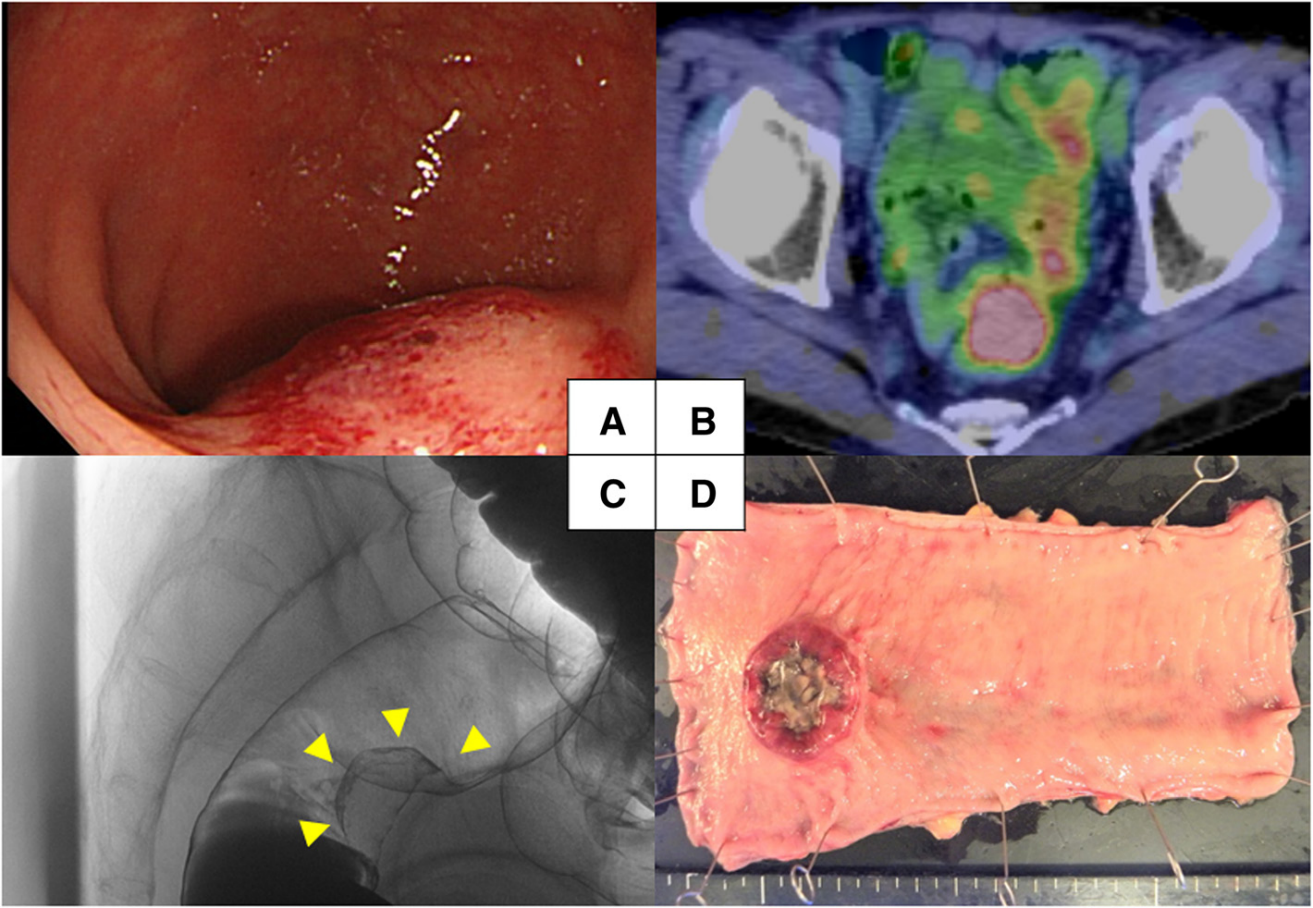
Preoperative findings and resected rectum specimen. **a** Colonoscopy image showing a 30-mm rectal mass that appears to be a submucosal tumor. **b** Fluorodeoxyglucose positron emission tomography-computed tomography image showing abnormally high uptake in part of the tumor and regional lymph nodes. **c** Contrast enema film showing deformation of the rectum. **d** Macroscopic appearance of the resected rectum specimen. There is no evidence of exposure of tumor or peritoneal dissemination

Two years after curative resection, a follow-up colonoscopy revealed a rectal mass; the patient was asymptomatic. The mass measured 30 mm in the greatest dimension and appeared to be a submucosal tumor (Fig. 2a). Histopathologic examination of an endoscopic, ultrasound-guided, fine-needle aspiration biopsy established a diagnosis of invasive adenocarcinoma with features similar to those of the GC resected 2 years previously. The serum level of carcinoembryonic antigen was slightly increased at 8.8 mg/mL, and fluorodeoxyglucose positron emission tomography-computed tomography showed high uptake in part of the rectal mass and regional lymph nodes (maximum standard uptake values being 8.8 and 3.2, respectively); however, there was no evidence of distant metastasis or peritoneal dissemination (Fig. 2b). Barium enema X-ray films showed the tumor at Ra-Rs (Fig. 2c). After a staging laparoscopy confirmed the absence of findings suggestive of serosal involvement or peritoneal dissemination, including negative peritoneal washing cytology, a laparoscopic low anterior resection with lymph node dissection was performed (Fig. 2d).

**Fig. 3:**
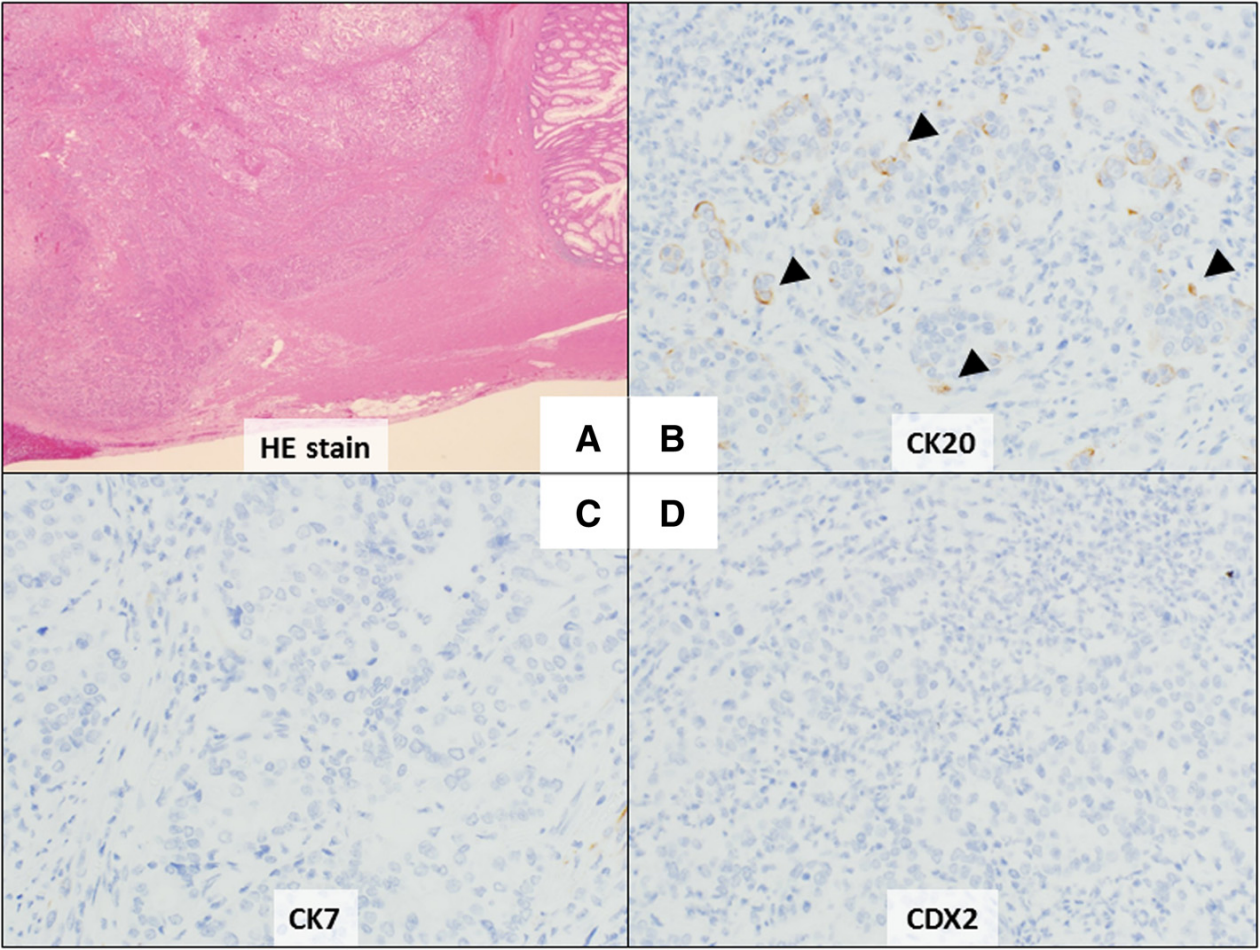
Analysis of rectal tumor by immunohistochemistry. **a** Photomicrograph showing moderately differentiated tubular adenocarcinoma mainly in the submucosal layer without serosal exposure (hematoxylin and eosin stain, ×12.5). **b**–**d** Immunohistochemical staining showed the tumor cells are positive for cytokeratin 20 and negative for cytokeratin 7 and caudal-type homeobox 2

Microscopically, the tumor was found to mainly be located in the submucosal layer and showed features of moderately differentiated tubular adenocarcinoma (Fig. 3a). Lymph node metastases were identified. There was lymphatic invasion (ly1) and venous invasion (v1). The serosal surface was free of disseminated tumor. Immunohistochemically, there were foci of carcinoma cells that were positive for cytokeratin 20 (Fig. 3b); however, they were negative for cytokeratin 7 (Fig. 3c). Negative staining for caudal-type homeobox 2, a transcription factor indicating goblet cell differentiation, combined with absence of intramucosal carcinoma in the rectal mucosa (Fig. 3d), suggested a diagnosis of metastatic adenocarcinoma of gastric origin. Morphologically, the tumor was similar to the third tumor excised. Because the peritoneal surface was unaffected both grossly and microscopically, the route of metastatic spread was considered to be hematogenous or lymphatic. The patient was alive with no evidence of recurrent tumor on imaging studies 6 months after the final surgery.

The recurrence patterns after curative surgery for GC have been classified as follows: (1) locoregional recurrence, (2) peritoneal recurrence, and (3) distant (including hematogenous) metastasis [3, 6, 9–11, 18]. The most common pattern of spread of these tumors is via peritoneal dissemination. As to distant metastases, intestinal metastasis from GC is rare; however, the most common cause of secondary neoplastic infiltration of the colon is GC [19]. Metastatic colonic lesions can occur by direct extension of the initial primary GC via the gastrocolic ligament, peritoneal metastasis, or hematologic or lymphatic dissemination [13, 19].

Hematologic or lymphatic spread from a GC to the rectum is extremely rare. We here report a metastatic rectal lesion with no associated peritoneal dissemination. To the best of our knowledge, there are only five English language reports of such cases (Table 1) [12–16]. Two of these were considered to have spread via the hematogenous route, whereas the route of spread in the other three cases was unclear. This is the first English language report of metachronous rectal metastasis from an “early” GC caused by hematologic or lymphatic metastasis. Although a perforation did occur during the second ESD in our case, there was no evidence of exposure of tumor, peritoneal dissemination, or cancerous ascites. Thus, the intraoperative and pathological findings suggested that the metastatic rectal lesion was attributable to hematologic or lymphatic metastasis rather than peritoneal dissemination. In addition, in all previous reports of rectal metastasis unassociated with peritoneal dissemination, the patients had advanced GC, whereas our case was categorized as T1aN0M0 stage IA by histological examination. Thus, our case indicates that even patients with “early” GC may develop rectal recurrence by hematologic or lymphatic spread without peritoneal dissemination.

**Table 1:** Literature reports of colorectal recurrence from gastric cancer

No.	Year	Author	Age	Sex	Histological type of GC	Stage (AJCC seventh edition)	Pattern of recurrence	Location	Lymph node metastasis	Histological type of recurrence	DFI(month)	Adjuvant	Outcome
1.	1994	Ogiwara et al.	53	F	por	NA	NA	D	Yes	por	660	Unknown	NA
2.	2009	Pace U et al.	77	M	por sig	TisN2Mx IIIB	NA	A	Yes	por sig	14	None	NA
3.	2011	Lim S W et al.	43	F	por	T4aN0M0 IIB	Hematologic	R	No	por	34	Present	36M alive
4.	2012	Tural D et al.	74	F	well	NA	Hematologic ?	R	NA	NA	–	No operation Chemotherapy	Alive
5.	2014	Sung Young Oh	69	F	por	T2N1M0 IIA	NA	S	NA	por	47	No operation Chemotherapy	Alive
6.	2015	Our case	63	M	well	T1aN0M0 IA	Hematologic or lymphatic	R	Yes	mod	28	None	6M alive

*Abbreviations*: *por* Poorly differentiated adenocarcinoma, *sig* Signet-ring cell carcinoma, *mod* moderately differentiated type, *A* ascending colon, *D* descending colon, *DFI* disease-free interval, *GC* gastric cancer, *NA* not available, *R* rectum, *S* sigmoid colon, *T* transverse colon

There are only a few published studies regarding surgical treatment of GC recurrence. According to several reports on the role and outcomes of surgical treatment for non-hepatic intra-abdominal recurrences from GC, surgical resection is the treatment of choice for selected patients in whom the recurrent tumors are completely resectable [20, 21]. Nunobe et al. reported the outcomes of surgery with curative intent in 36 selected patients with locoregional recurrence. Their median survival after surgery was about 23 months, seven of the 36 patients (19.4 %) surviving more than 3 years after surgery [22]. In the present case, the factors favoring curative surgical resection were that there was no evidence of exposure of tumor or peritoneal dissemination, the recurrence had appeared late and was isolated, and the intra-abdominal recurrent tumor could be completely resected en bloc. Interestingly, our case confirms what others have reported: that metastatic lesions may have regional lymphatic metastases like primary rectal cancer (Table 1). Ogiwara et al. reported a case of polypoid colonic metastases with regional lymph node metastases 11 years after the resection of a GC [12]. Additionally, Pace et al. reported a case of a signet ring cell carcinoma of the stomach metastasizing to the ascending colon with regional lymph node metastases [13]. Therefore, it is necessary to investigate for possible lymphatic metastases preoperatively and perform systematic lymph node dissection if indicated when resecting a recurrent tumor.

In the recent studies, a local recurrence rate after curative ESD for GC is generally low (in the range 0.1–1.1 %) [23, 24]. On the other hand, a higher incidence of metachronous GCs (in the range 1.8–15.9 %) has been reported [24–27]. In the present case, all three GCs were located at the similar part, the anterior wall of the fornix. However, the pathological records of the first and second ESDs demonstrated that those ESDs were performed curatively, and three tumors did not have any specific characteristics. Considering the rates of local recurrence and metachronous GC, it is extremely difficult to consider that local recurrence occurred twice after curative ESDs. Rather, it is possible that metachronous GCs occurred twice after curative ESD at the similar part.

In summary, we found the tumor at the rectum which appeared to be a submucosal tumor, and there were no findings of serosal exposure of tumor, peritoneal dissemination, and cancerous ascites. These findings support that the metastatic rectal lesion was due to hematologic or lymphatic dissemination, but not peritoneal metastasis. In our case, at least two distinct types of GCs, namely a gastric type and a Lauren’s intestinal type, were identified by gastrectomy and ESD, respectively. However, which type of GC caused rectal metastasis was obscured. Importantly, we confirmed that a rectal tumor was derived from an intramucosal GC, which is an extremely rare event.

## Conclusions

We here report a case of rectal metastasis caused by hematologic or lymphatic metastasis 2 years after curative resection for “early” GC. Although rectal metastasis from GC, particularly when attributable to hematologic or lymphatic metastasis, is very rare, metastatic gastric adenocarcinoma should be considered as a differential diagnosis for patients who present with a rectal tumor and a past history of GC, even if it is an early GC.

## Consent

Written informed consent was obtained from the patient for publication of this case report and any accompanying images. A copy of the written consent is available for review by the Editor-in-Chief of this journal.

## Abbreviations

ESD, endoscopic submucosal dissection; GC, gastric cancer
